# Should “on-demand” treatments for Parkinson’s disease OFF episodes be used earlier?

**DOI:** 10.1016/j.prdoa.2022.100161

**Published:** 2022-08-12

**Authors:** Stuart H. Isaacson, Fernando L. Pagan, Mark F. Lew, Rajesh Pahwa

**Affiliations:** a Parkinson’s Disease and Movement Disorders Center of Boca Raton, 951 NW 13th St, Bldg. 5-E, Boca Raton, FL 33486, USA; b Medstar Georgetown University Hospital, 3800 Reservoir Road NW, 7^th^ Floor, PHC Building, Washington, DC 20007, USA; c Keck School of Medicine of USC, HCC2, Ste 3000, 1520 San Pablo Street, Health Sciences Campus, Los Angeles, CA 90033, USA; d University of Kansas Medical Center, 3599 Rainbow Blvd, Kansas City, KS 66103, USA

**Keywords:** Rescue therapy, Motor fluctuations, Apomorphine sublingual film, Apomorphine hydrochloride injection, Levodopa inhalation powder

## Abstract

We discuss a shift in the treatment paradigm for OFF episode management in patients with Parkinson’s disease, based on clinical experience in the United States (US). Three “on-demand” treatments are currently available in the US as follows: subcutaneous apomorphine, levodopa inhalation powder, and sublingual apomorphine. We empirically propose that “on-demand” treatments can be utilized as a complementary treatment when OFF episodes emerge and can be utilized when needed rather than reserving these treatments only until other treatment approaches (adjustment of baseline treatment and/or addition of adjunctive treatment with “ON-extenders”) have failed. Current treatment approaches combine “ON-extenders” with increasing levodopa dosing and/or frequency to treat OFF episodes. Yet, OFF episodes often persist, with a substantial amount of daily OFF time. OFF episode treatment is hindered by variable gastrointestinal (GI) absorption of oral levodopa, reflecting GI dysmotility and protein competition. Novel “on-demand” treatments bypass the gut and can improve OFF symptoms more rapidly and reliably than oral levodopa. With the emergence of novel “on-demand” treatments, we conclude that a shift in treatment paradigm to the earlier, complementary use of these medications be considered.

## Highlights

• OFF episodes are common in patients as Parkinson’s disease progresses.
• OFF episodes are typically managed with “ON-extenders” and changes in levodopa dosing.
• OFF episodes persist despite conventional treatment.
• “On-demand” therapies can rapidly improve OFF symptoms.
• A shift to earlier complementary use of “on-demand” therapies should be considered.

## 1 Introduction

### 1.1 Background: prevalence, types, and causes of OFF episodes

Parkinson’s disease (PD) is characterized by motor and nonmotor symptoms reflecting widespread synuclein aggregation and resulting degeneration of dopaminergic neurons in the substantia nigra pars compacta [1]. In addition, the development of PD also involves widespread pathophysiology involving cholinergic and other monoamine systems [2]. Progressive loss of dopaminergic neurons in the substantia nigra leads to motor symptoms, and treatment with oral levodopa (with carbidopa or benserazide) initially provides rapid, robust and long-duration benefit [2]. However, as the disease progresses, the consistent symptom benefit of each dose of oral levodopa wanes [2], [3]. This diminished effect of a levodopa dose has been termed an OFF episode, and these fluctuate with benefit (or ON) of subsequent doses [4]. OFF episodes appear within 1–2 years in some patients, by 5 years in 50 % of patients, and in most patients beyond 9 years [5], [6]. OFF episodes continue to be present despite daily adjunctive medications and increasing dose and frequency of levodopa [7], [8], [9], [10], [11], [12], [13], [14].

An OFF episode may be heralded by the gradual return of nonmotor symptoms or mild motor symptoms, or the return of symptoms may be abrupt [4]. OFF episodes can vary in type and severity, and are often unpredictable [15]. Common motor symptoms can include resting tremor, bradykinesia, rigidity, hypophonia, dystonia, and gait dysfunction/postural instability [1], [2], [4]. Nonmotor symptoms can include anxiety, bradyphrenia, pain and sensory disturbances, autonomic dysfunction, apathy, and fatigue [2], [4]. During an OFF episode, some patients may be mildly impaired, while others can be completely disabled, significantly impacting daily activities and quality of life [16]. In a Michael J. Fox Foundation survey of > 3000 patients with PD, >90 % of respondents reported ≥ 1 OFF episode per day, nearly 50 % reported OFF episodes as having a moderate to severe effect on daily life, and 44 % reported a moderate to severe effect on their health and well-being [17]. OFF episodes also contribute to a greater burden to the health care system through increased emergency department visits and hospitalizations [18].

Morning akinesia may be the first emergence of motor fluctuations, occurring upon awakening when there is no longer benefit of the prior day’s last dose of levodopa, and before onset of the first daily dose [4]. Morning akinesia is common, with an estimated prevalence of ∼ 60 %, and impacts quality of life [4], [19], [20]. OFF episodes occurring in the morning may be prolonged when the onset of the first daily levodopa dose is delayed (delayed ON) or fails to provide symptom benefit (dose failure or no ON) [4]. In one open-label study of patients with PD and morning akinesia, the mean time to ON after a levodopa dose was 61 min [21]. In a single-visit pilot study of patients with PD on stable doses of levodopa for ≥ 4 weeks, 51 % of those experiencing motor fluctuations reported delayed time to ON after their first daily dose of levodopa [22]. Additionally, 21 % reported having delayed ON every morning, and 14 % reported having ≥ 1 dose failure in a 1-week period [22]. Morning akinesia occurred despite adjunctive treatment added to levodopa (85 % on dopamine agonists, 50 % on catechol-O-methyltransferase [COMT] inhibitors, and 92 % on monoamine oxidase-B [MAO-B] inhibitors) [22]. Delayed ON may be a major contributor to the total daily OFF time experienced by patients. In a study of patients with advanced PD, mean time to ON (46 ± 21 min) was more than double the duration of wearing OFF (21 ± 14 min) for a single dose of levodopa [23]. Overall, delayed ON comprised 68 % of total daily OFF time [23].

In addition to morning akinesia, OFF episodes can occur throughout the day [4]. End-of-dose wearing OFF of benefit is common [24] and can often be anticipated by a patient. Other OFF times may be unexpected, such as delayed ON, suboptimal ON, dose failure, and unpredictable OFF, which is uncommon and occurs when patients rapidly shift from ON (with or without dyskinesia) to OFF without warning [2], [4], [22]. Unpredictable OFF is thought to be related to postsynaptic pharmacodynamic changes, whereas end-of-dose wearing OFF typically reflects the relatively short pharmacokinetic plasma levodopa half-life and the progressive loss of presynaptic striatal dopamine buffering capacity [2], [4].

In contrast to the previously described OFF episodes, delayed ON and dose failure reflect variability in the absorption of oral levodopa [4]. Dysphagia may be common in patients with PD and can lead to residual oral antiparkinsonian drugs in the pharynx [25]. Levodopa is mainly absorbed via active transport by large neutral amino acid transporters in the proximal small intestine [4]. Many factors can impair or delay levodopa transport from the gut lumen into the circulation. Delayed delivery to the intestine due to esophageal dysmotility and gastroparesis can lead to variable and delayed delivery to the proximal small intestine [4], [26]. Dietary amino acids can compete with levodopa for transport across the intestinal lumen and for transport across the blood–brain barrier by the large neutral amino acid transporter [4]. Small bowel bacterial overgrowth, *Helicobacter pylori* infection, and metabolism of levodopa by gut bacterial microbiota can also impede transport of levodopa from the intestine into the plasma and brain [26], [27]. Finally, extensive enzymatic breakdown of levodopa by human aromatic l-amino acid decarboxylase and COMT occurring in gastrointestinal (GI), muscle, and peripheral tissues can significantly reduce levodopa bioavailability [28], [29].

## 2 Therapeutic dilemma: Current strategy to treat OFF episodes may be suboptimal

**Fig. 1:**
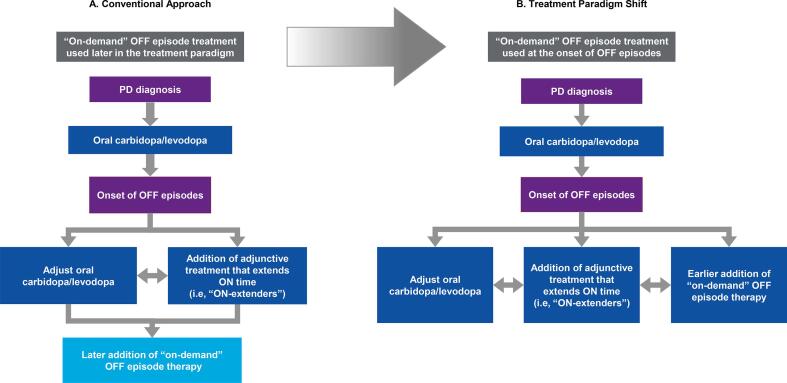
Two pharmacologic approaches to manage OFF episodes in patients with PD. (A, Conventional Approach; B, Treatment Paradigm Shift) PD, Parkinson’s disease.

A common therapeutic dilemma evolves in managing OFF episodes while patients are on oral antiparkinsonian treatment regimens. Strategies that feature prominently in guidelines [30], [31] or published algorithms [1], [32] typically include 1 of 2 conventional approaches (Fig. 1A). The first is to adjust the current levodopa regimen by altering the dose and/or dosing frequency or switching to an extended-release formulation [1], [32]. The second conventional approach is to add adjunctive treatment (herein, called “ON-extenders”) to lengthen the duration of ON time [1], [30], [31], [32]. Unfortunately, many patients continue to experience OFF episodes despite the adjustment of levodopa and treatment with “ON-extenders” [21], [22].

“ON-extenders” have predominantly been demonstrated to prolong the duration of ON time, whereas their effect related to initiating ON is understudied. Only mild improvements in turning ON were found with pramipexole or entacapone [33], [34]. In contrast, non-GI “on-demand” treatments can be used complementary to or between doses of levodopa to turn patients ON more rapidly and reliably when oral levodopa onset is variable. However, this therapeutic approach has not been routinely discussed in previous guidance from professional societies or in the literature. Instead, “on-demand” treatment is typically considered to address OFF episodes only well after adjustment of baseline levodopa and/or adding “ON-extenders” (Fig. 1A) [1], [32].

Several United States Food and Drug Administration (FDA)–approved “on-demand” treatments are available (Table 1) as follows: apomorphine hydrochloride injection (APOKYN®; Supernus Pharmaceuticals, Inc; Rockville, MD), administered with a pen injector device; levodopa inhalation powder (INBRIJA®; Acorda Therapeutics, Inc; Ardsley, NY) administered with an inhaler device; and apomorphine sublingual film (KYNMOBI®; Sunovion Pharmaceuticals Inc.; Marlborough, MA) [35]. Apomorphine hydrochloride injection is also approved outside of the United States (APO-go®; Britannia Pharmaceuticals Limited; Berkshire, UK; MOVAPO™; Paladin Labs Inc.; Quebec, CA) [36], [37]. These “on-demand” treatments can be self- or caregiver-administered during an OFF episode. Because they bypass GI dysmotility (and other intestinal absorption-related factors), they can reliably and rapidly switch patients from OFF to ON, effectively shortening the OFF episode duration [38], [39], [40].

**Table 1:** Approved “on-demand” treatments for OFF episodes [38], [39], [40], [57], [63].

Drug name	FDA approval/development phase	Dosing^a^	Mean change in UPDRS Part III scores in pivotal study (active drug vs placebo)
Apomorphine hydrochloride injection (APOKYN®) [38], [63]	Approved 2004	2–20 mg	20 min postdose^b^: −23.9 vs −0.1 (p < 0.001) Mean dose of active drug = 5.4 mg
Levodopa inhalation powder (INBRIJA®) [39], [57]	Approved 2018	Maximum 84 mg/OFF period	30 min postdose at Week 12:−9.8 vs −5.9 (p = 0.0088) Randomized dose of active drug = 84 mg
Apomorphine sublingual film (KYNMOBI®) [35], [40], [63]	Approved 2020	10–30 mg	30 min postdose at Week 12:−11.1 vs −3.5 (p = 0.0002)^c^

FDA, United States Food and Drug Administration; UPDRS, Unified Parkinson’s Disease Rating Scale; MDS, Movement Disorder Society.

a Treatments used “on-demand” up to 5 times daily.

b Endpoint was measured during inpatient phase of unspecified duration.

c Measurement was for MDS-UPDRS Part III.

Based on our clinical experience, we review and discuss advantages and disadvantages of a treatment paradigm shift to an earlier, complementary use of “on-demand” treatments (i.e., adding “on-demand” treatment at the same time as, or instead of, adjusting oral levodopa and/or adding an “ON-extender”) to help manage OFF episodes in patients with PD (Fig. 1B) versus addressing OFF with the conventional therapeutic approach (i.e., late addition of “on-demand” treatments only after adjusting oral levodopa and/or adding an “ON-extender”; Fig. 1A).

## 3 Current (conventional) approach to manage OFF episodes (use of “ON-extenders”)

There are more than 15 antiparkinsonian treatments available for patients with PD in the United States that can extend the duration of an ON period and lessen daily OFF time (Table 2). Immediate, controlled, and extended-release formulations of levodopa help facilitate flexibility in dose adjustment and frequency. Immediate-, extended-, and transdermal-release dopamine receptor agonists can be used to reduce daily OFF time. Selective MAO-B inhibitors can reduce striatal dopamine metabolism [2]. Peripheral COMT inhibitors can prolong plasma levodopa levels, allowing greater entry into the brain [2]. Non-dopaminergic antagonists to adenosinergic and glutamatergic receptors can also reduce daily OFF time [2].

**Table 2:** Summary of daily dosing of Parkinson’s disease treatments.^a^

Antiparkinsonian medications	Daily dosing, range (mg)	Daily dosing frequency, range	Year approved
Carbidopa/levodopa			
IR (SINEMET®)	30/300– 200/2000	3–8	1975
CR (SINEMET®)	100/400– 400/1600	2–6	1991
ODT (PARCOPA®)	30/300– 200/800	3–4	2004
Carbidopa/levodopa/entacapone (STALEVO®)	12.5/50/200–300/1200/1600		2003
ER (RYTARY®)	71.25/285–612.5/2450	3–5	2015
Dopamine agonists			
Pramipexole (MIRAPEX®)	0.375–4.5	3	1997
Pramipexole ER (MIRAPEX ER®)	0.375–4.5	1	2010
Ropinirole (REQUIP®)	0.75–24	3	1997
Ropinirole (REQUIP® XL)	2–24	1	2008
Rotigotine (NEUPRO®) transdermal	2–8	1	2007
Monoamine oxidase-B inhibitors			
Rasagiline (AZILECT®)	0.5–1	1	2006
Selegiline (ZELAPAR®) ODT	1.25–2.5	1	2006
Safinamide (XADAGO®)	50–100	1	2017
Catechol-O-methyltransferase inhibitors			
Tolcapone (TASMAR®)	300–600	3	1998
Entacapone (COMTAN®)	200–1600	1–8	1999
Opicapone (ONGENTYS®)	50	1	2020
Additional			
Amantadine ER (GOCOVRI®)	137–274	1	2017
Istradefylline (NOURIANZ™)	20–40	1	2019

IR, immediate release; CR, continuous release; ODT, orally disintegrating tablet; ER, extended release.

a Data are from United States package inserts.

Numerous pivotal trials have evaluated the efficacy of different formulations of levodopa and “ON-extenders” to reduce daily OFF time, suggesting these approaches can reduce OFF time by 9–42 % (e.g., ∼6 h at baseline to ∼4 h at endpoint; Table 3). In addition to efficacy, there are proven quality of life benefits associated with conventional treatments for PD [11], [41], [42], [43]. Levodopa and the fixed-dose combination of carbidopa/levodopa with entacapone have all been associated with improved quality of life across multiple scales, including Parkinson’s Disease Questionnaire (PDQ-39), EQ-5D, Patient Global Impression of Change, and the modified Rankin scale for neurologic disability [41], [42]. Similarly, the dopamine agonists pramipexole and ropinirole have both been associated with improved scores for PDQ-39, EQ-5D, and the Beck Depression Inventory®-II [11], [43].

**Table 3:** Summary of reduction in OFF time for Parkinson’s disease treatments.^a^

Medication	Baseline daily OFF time	OFF time reduction (difference vs placebo)
Carbidopa/levodopa formulations		
IR/ER/+ entacapone	5.9–6.8 h	1.0–2.2 h/day^b^
Dopamine agonists		
Pramipexole/ropinirole	6.0–6.4 h	0.6–2.0 h/day
Pramipexole ER/ropinirole XL/rotigotine	6.3–7.0 h^c^	0.7–1.8 h/day
Monoamine oxidase-B inhibitors		
Rasagiline/safinamide/selegiline ODT	5.4–7.0 h	0.8–1.6 h/day
Catechol-O-methyltransferase inhibitors		
Entacapone/opicapone	6.2–6.8 h	0.9–1.0 h/day
Additional		
Amantadine ER [72], [73]	2.6–3.2 h	0.8–1.1 h/day
Istradefylline	6.0–6.6 h	0.7–0.9 h/day

IR, immediate release; ER, extended release; XL, extended release; ODT, orally disintegrating tablets.

a Representative but not exhaustive list of currently available treatments; data are from United States package inserts, unless otherwise referenced.

b One study in the range did not report OFF time reductions versus placebo.

c Baseline daily OFF time was not reported for pramipexole ER.

Despite dose adjustments of levodopa and treatment with several “ON-extenders” from different classes, OFF episodes persist for many patients [44], [45]. Data from clinical trials suggest that patient OFF time can range from ∼3 to 7 h per day, and dopamine agonists or other “ON-extenders” added on to levodopa only offer ∼0.6–2.0 h per day of decreased net OFF time (Table 3). In one study, patients experienced an average of 7 h of daily OFF time, in which addition of ropinirole only decreased total OFF time by 2.1 h [11].

Although some degree of improvement in OFF time can be achieved with adjusting levodopa and adding “ON-extenders,” it is not without potential drawbacks or adverse events (AE). Increasing daily doses of levodopa may increase dyskinesia [46]. Increasing dosing frequency may reduce adherence and require adjustment of mealtimes to lessen protein competition with levodopa [4], [47], potentially leading to unintended calorie restriction and weight loss. In addition, inclusion of “ON-extenders” to levodopa may increase medication noncompliance and side effects [47], [48].

Scheduled doses of levodopa with or without “ON-extenders” may be a preferred treatment approach for some patients with PD, as research suggests there may be difficulty for the patient in recognizing OFF episodes, and thus, uncertainty regarding when to use “on-demand” treatment. In a survey of patients with PD and their physicians and caregivers, patients identified variability of OFF symptoms, difficulty describing symptoms, and the perception that OFF episodes cannot be improved as major barriers to treatment [49]. Movement disorder specialists likewise identified patient difficulty in recognizing OFF symptoms but also identified poor understanding of OFF episodes and their relationship to timing of drug treatment and cognitive impairment of patients as additional barriers [49].

## 4 Use of “on-demand” treatments to treat OFF episodes

Previous attempts to treat delayed ON by reducing time to ON of levodopa have been made. Since liquids empty through the pylorus into the small intestine quicker than solids, liquid levodopa has been tried [50], but hourly administration often leads to discontinuation for most patients. Dispersible levodopa formulated for absorption in the lower GI tract is available for use outside the United States. An initial trial demonstrated improvement in time to ON compared with standard oral levodopa [51], while 2 subsequent studies failed to replicate these results, without any improvement in time to ON [52], [53]. Subcutaneous methyl ester levodopa also failed to significantly shorten time to ON [54]. Taking an additional levodopa tablet, moving up the time of the next dose, chewing oral levodopa tablets, or taking levodopa with carbonated beverages have anecdotally been reported to have quicker onset, but have not been studied; as all of these formulations remain as solids unless completely dissolved in solution, gastric emptying would still be delayed [55]. The use of “on-demand” treatments that are not absorbed in the gut can avoid the GI variability of response that occurs with oral levodopa.

The “on-demand” treatment with the longest clinical experience to date and used globally for decades is apomorphine hydrochloride injection, approved by the FDA in 2004 (Table 1). In a pivotal trial, the Unified Parkinson’s Disease Rating Scale (UPDRS) motor score was significantly improved from baseline versus placebo (–23.9 vs –0.1; p < 0.001) with a mean time to onset of 22 min, and onset within 8 min as assessed subjectively by patients [38], [56]. Levodopa inhalation powder was approved by the FDA in 2018 on the basis of a pivotal trial, which showed significant improvement in UPDRS motor score at 30 min postdose versus placebo (−9.8 vs −5.9; p = 0.0088), where 58 % of patients achieved an ON response within 60 min, and onset of motor score benefit at 10 min [39], [57]. Apomorphine sublingual film was FDA approved in 2020 [35]. In a pivotal study, a significant reduction in Movement Disorder Society (MDS)-UPDRS Part III score at 30 min postdose versus placebo was observed at Week 12 (−11.1 vs −3.5; p = 0.0002) and 35 % of patients achieved FULL ON within 30 min, and onset of motor score benefit at 15 min [40]. Apart from AEs that are dopaminergic class effects (e.g., nausea, vomiting, somnolence, etc.), “on-demand” treatment is also associated with AEs unique to the respective formulation/delivery system (apomorphine hydrochloride injection: injection site reactions; levodopa inhalation powder: cough, upper respiratory tract infections, and sputum discoloration; apomorphine sublingual film: oral/pharyngeal soft tissue swelling, pain, and paresthesia).

These “on-demand” treatments bypass the GI variability of levodopa absorption and have a rapid and reliable onset for treatment of OFF episodes. The time to reach maximum plasma concentration (T_max_) of “on-demand” treatments can be shorter than the T_max_ of levodopa, which may make them well suited for treatment of OFF episodes, consisting of delayed ON. Data directly comparing apomorphine formulations to initial morning doses of levodopa demonstrate that apomorphine has an equivalent clinical benefit with faster onset. In 2 studies comparing apomorphine hydrochloride injection with levodopa, motor responses were similar between treatments, but faster onset was observed with apomorphine (3–14 vs 19–75 min) [58], [59]. In an open-label study, patients with morning OFF with delayed onset of oral levodopa benefit were treated with apomorphine hydrochloride injection and experienced a reduction from baseline of 37 min in mean time to ON [21]. In addition, patient-assessed onset of ON within 60 min postdose was achieved on more days treated with apomorphine hydrochloride injection versus levodopa (93 % vs 54 %, respectively) [21]. In a post hoc analysis of the pivotal study of apomorphine sublingual film, the magnitude of motor response during open-label dose titration was ∼ 2-fold higher for sublingual film versus the first morning levodopa dose at 15 min postdose, and the peak response occurred earlier (45 vs 90 min) [60]. Further, patient-reported FULL ON was achieved by 72 % of patients treated with apomorphine sublingual film within 30 min postdose [60].

## 5 Changing the treatment paradigm to earlier use of “on-demand” treatments?

The approach of introducing “on-demand” treatment as a complementary therapy earlier in the treatment paradigm may benefit patients. OFF episodes often persist and/or recur despite adjustment of levodopa doses and the addition of adjunctive “ON-extender” treatments [61]. When the benefit of the last oral levodopa dose wanes, an OFF episode will occur until the onset of benefit from the next levodopa dose [4]. Owing to the variability of onset of oral levodopa doses, OFF episodes can be prolonged [4]. Based on our clinical experience, use of an “on-demand” treatment when needed can empower patients to return to ON more rapidly and reliably.

Other potential benefits of earlier complementary use of “on-demand” treatments include less frequent adjustment of the baseline pharmacotherapy regimen, less frequent dosing intervals, and fewer medication times each day. Patients may also feel greater confidence in performing daily activities without unexpected disruptions. This may be especially important in cases when OFF episodes are infrequent but severe. This methodology mirrors a successful treatment approach employed in the management of migraines, in which baseline disease management is supplemented with rapid “on-demand” treatment [62]. Finally, “on-demand” treatments for PD may be useful with advanced therapies, such as before or after deep brain stimulation surgery, or with continuous infusion of intrajejunal levodopa enteral suspension or subcutaneous infusion of apomorphine or levodopa.

“On-demand” medications are approved for the treatment of up to 5 OFF episodes per day [35], [57], [63]. Initially, a patient can choose to use an “on-demand” treatment to reverse an OFF episode that occurs predictably during the day (e.g., early morning akinesia). Patients can then begin to use the medication when needed to treat OFF episodes that occur at other times of the day (end-of-dose wearing OFF) or unexpectedly (e.g., delayed ON, dose failure). “On-demand” treatments are most useful for patients who can self-identify when they are having OFF episodes and correspondingly treat their symptoms. The ability to recognize OFF may be addressed through patient education [49]; however, patient self-recognition of OFF episodes and the relation between medication timing and OFF episodes remains a major treatment barrier [49], [64]. Observations from pivotal trials of “on-demand” treatments point to a current pattern of underutilization despite patient education in the clinical trial setting (“on-demand” treatment use ∼2 times daily compared with ∼4 OFF episodes per day in baseline diaries) [38], [39], [40]. Additionally, patients note that the need for “on-demand” treatment may change daily, which may account for a lower number of “on-demand” treatment administrations compared with OFF episode frequency.

Barriers to “on-demand” treatment can limit use. Training on use of the levodopa inhaler device and the subcutaneous apomorphine injection pen and how to properly use apomorphine sublingual film (drinking water to moisten the mouth, placing the entire film under the tongue, and allowing the film to completely dissolve without swallowing for 3 min) may be needed to ensure successful administration. Some patients with severe motor OFF episodes can have difficulty preparing and using the pulmonary inhaler or injection pen. The 2 apomorphine formulations require dose optimization with direct observation of the initial dose and guidance to identify an optimal dose that mimics the ON they experience with levodopa with rapid onset and tolerability for each patient [35], [63]. Once identified, this optimized dose usually does not require change during ongoing use. Pretreatment with an antiemetic before apomorphine initiation is recommended in the prescribing information for both formulations [35], [63]. Domperidone is used in Europe, but is not available in the United States [65], [66]. Trimethobenzamide is used in the United States [67], when supply is available, but evidence of efficacy in reducing nausea and vomiting during treatment initiation is lacking [68], [69]. One study found significantly reduced nausea and vomiting for the first 2 months after initiation, but did not observe a significant difference on Day 1 (primary endpoint) or during Month 3 of treatment [68]. Other antiemetics should not be used because they either block dopamine receptors and worsen parkinsonism, or block 5HT_3_ receptors, causing severe hypotension [35], [63]. Cough is the most common AE of levodopa inhalation powder [57], probably reflecting an irritant effect of the dry levodopa powder [39]; this can limit its use, although patients may adjust force of inhalation to reduce cough [70]. Injection site reactions and nodules can limit use of subcutaneous apomorphine injections [38], [63]. Apomorphine sublingual film use may be limited by oral/pharyngeal soft tissue swelling, pain, and paresthesia [35].

## 6 Discussion: Shift of the paradigm is needed

Baseline demographics from clinical trials suggest that patients on oral levodopa can have up to 7 h of daily OFF time, and after addition of an “ON-extender” to baseline levodopa, may still have up to 5 h of daily OFF time (Table 3). Our focus over the past 3 decades has placed an emphasis on trying to provide more continuous dopaminergic therapies to reduce the occurrence of OFF as PD progresses [71]. This approach implies multiple adjustments of levodopa dosing and frequency combined with “ON-extender” treatment. Yet OFF persists, and AEs often limit treatment over the course of the patient’s journey [1], [2].

It is thus of more than passing interest to consider the complimentary use of “on-demand” treatments earlier in the treatment paradigm. Empowering patients with therapies that can offer a reliable and rapid ON may be preferable for some patients and should be discussed as part of the shared clinical decision-making when OFF episodes emerge. This could allow for the initial management of predictable OFF, with the ability to also use for unexpected OFF too. As with other antiparkinsonian medications, the benefits of treating OFF with “on-demand” treatments need to be balanced against the potential risks, and the potential risks need to be weighed against the possibility of leaving patients with OFF time that is inadequately treated and addressed.

## 7 Conclusions

OFF episodes occur frequently, despite higher levodopa dose and adjunctive treatments. “On-demand” treatment can be used when needed (up to 5 times per day) to provide rapid and reliable return to ON, without changes to the existing regimen of antiparkinsonian medications. Patient education regarding OFF episode recognition and safe use of complementary “on-demand” treatments may help to improve awareness of the utility of these treatments, which have been largely underutilized to date.

A treatment paradigm shift to consider “on-demand” treatments earlier and throughout the disease course is supported by the persistence of OFF despite adjunctive treatment, the emerging understanding of GI dysmotility and variability of oral levodopa absorption, and the impact of OFF on daily activities and quality of life measures. We suggest that these “on-demand” treatments can empower patients to recognize and rapidly treat OFF episodes when they occur. Shared clinical decision-making should routinely incorporate these complementary “on-demand” treatments as a therapeutic option when OFF episodes emerge.

## Funding

This work was supported by funding from 10.13039/100009655Sunovion Pharmaceuticals Inc. (Marlborough, MA, USA).

## CRediT authorship contribution statement

**Stuart H. Isaacson:** Conceptualization, Supervision, Writing – original draft, Writing – review & editing. **Fernando L. Pagan:** Conceptualization, Supervision, Writing – original draft, Writing – review & editing. **Mark F. Lew:** Conceptualization, Supervision, Writing – original draft, Writing – review & editing. **Rajesh Pahwa:** Conceptualization, Supervision, Writing – original draft, Writing – review & editing.

## Declaration of Competing Interest

The authors declare the following financial interests/personal relationships which may be considered as potential competing interests: SHI reports honoraria for CME, consultant, research grants, and/or promotional speaker on behalf of AbbVie, Acadia Pharmaceuticals Inc., Acorda Therapeutics, Inc., Adamas Pharmaceuticals, Inc., Addex Therapeutics, AFFiRiS AG, Alexza Pharmaceuticals, Allergan, Amneal Pharmaceuticals LLC, Aptinyx Inc., Axial Therapeutics, Inc., Benevolent, Biogen, Biovie, Britannia Pharmaceuticals Ltd, Cadent Therapeutics, Cala Health, Cerecor Inc., Cerevel Therapeutics, Eli Lilly, Enterin Inc., GE Healthcare, Global Kinetics Pty Ltd, Impax Laboratories, Impel NeuroPharma, Intec Pharma, Jazz Pharmaceuticals, Kyowa Kirin, Lundbeck, Merz Pharmaceuticals, Michael J. Fox Foundation, Neuralys Inc, Neurocrine Biosciences, Inc., Neuroderm, Novartis, Parkinson Study Group, Pharma Two B Ltd., Praxis, Prilenia Therapeutics, Revance, Roche, Sage, Sanofi, Scion, Scion Neurostim, Stoparkinson, Sunovion Pharmaceuticals Inc., Sun Pharma, Supernus Pharmaceuticals, Inc., Teva Pharmaceuticals, Theravance Biopharma, Transposon, and UCB. FLP reports fees for consulting or speaker services from AbbVie, Acorda Therapeutics, Inc., Adamas Pharmaceuticals, Inc., Amneal Pharmaceuticals LLC, Kyowa Kirin, Merz Pharmaceuticals, LLC, Neurocrine Biosciences, Sunovion Pharmaceuticals Inc., Supernus Pharmaceuticals, Inc., Teva Pharmaceuticals, and US WorldMeds. He has received stock or has an ownership interest in KeifeRx and has received research support from Novartis and US WorldMeds. MFL reports consultant or speaker services from Acadia, Acorda Therapeutics, Adamas, Kyowa, Neurocrine, and Supernus Pharmaceuticals, Inc., and is an active researcher for Michael J. Fox Foundation, Neuraly Inc, NIAA, Parkinson Study Group, Pharma Two B Ltd., and Sun Pharma. RP reports honoraria, consultant fees, and/or research grants from Abbott Laboratories, AbbVie, Acadia Pharmaceuticals Inc., Acorda Therapeutics, Inc., Adamas Pharmaceuticals, Inc., Amneal Pharmaceuticals LLC, Biogen, Biohaven Pharmaceuticals Inc., Boston Scientific, Cala Health, EIP Pharma, Inc., Eli Lilly, Global Kinetics Pty Ltd, Kyowa Kirin, Lundbeck, Mitsubishi Tanabe Pharma America, Neuraly Inc., Neurocrine Biosciences, Parkinson Foundation, Pharma Two B Ltd., Prilenia Therapeutics, Roche, Sage Therapeutics, Sun Pharma, Sunovion Pharmaceuticals Inc., Theranexus, Theravance Biopharma, US WorldMeds LLC, and Voyager.
